# Dominance status predicts social fear transmission in laboratory rats

**DOI:** 10.1007/s10071-016-1013-2

**Published:** 2016-07-13

**Authors:** Carolyn E. Jones, Marie-H. Monfils

**Affiliations:** Department of Psychology, The University of Texas at Austin, 108 E. Dean Keeton Stop A8000, Austin, TX 78712-1043 USA

**Keywords:** Social learning, Fear conditioning, Dominance, Play behavior

## Abstract

Acquiring information about stimuli that predict danger, through either direct experience or inference from a social context, is crucial for individuals’ ability to generate appropriate behaviors in response to threats. Utilizing a modified demonstrator–observer paradigm (fear conditioning by proxy) that allows for free interaction between subjects, we show that social dominance hierarchy, and the interactive social behaviors of caged rats, is predictive of social fear transmission, with subordinate rats displaying increased fear responses after interacting with a fear-conditioned dominant rat during fear retrieval. Fear conditioning by proxy conserves some of the pathways necessary for direct fear learning (e.g., lateral amygdala) but is unique in that it requires regions necessary for emotional regulation (e.g., anterior cingulate cortex), making this paradigm an important tool for evaluating learning and behavior in the laboratory setting.

**Electronic supplementary material:**

The online version of this article (doi:10.1007/s10071-016-1013-2) contains supplementary material, which is available to authorized users.

## Introduction

The ability to learn about threatening events through direct experience with aversive stimuli has been extensively studied in several species, and the physiological pathways are well categorized (Maren 2001). First-hand encounter with a threat, however, is not the only way individuals learn about danger. A number of studies have described vicarious fear learning in both human (Hygge and Öhman 1978; Olsson and Phelps 2004) and non-human primates (Cook and Mineka 1987; Mineka and Cook 1993; Mineka et al. 1984), usually by way of observational fear learning; yet, literature on rodents’ ability to learn about danger indirectly through observing another’s reaction has only recently started to gain traction (Atsak et al. 2011; Bredy and Barad 2009; Bruchey et al. 2010; Guzmán et al. 2009; Jeon et al. 2010; Jones et al. 2014; Kavaliers et al. 2005; Kim et al. 2010; Knapska et al. 2010; Langford et al. 2006; Masuda et al. 2009; Pereira et al. 2012).

To date, most rodent models of social fear learning involve an animal that visually observes a fear reaction in a conspecific through a physical barrier (Atsak et al. 2011; Jeon et al. 2010; Kavaliers et al. 2005; Pereira et al. 2012) but see also (Kim et al. 2010). In laboratory and deer mice, social factors including familiarity (Jeon et al. 2010; Jones et al. 2014; Kavaliers et al. 2005), kinship (Kavaliers et al. 2005), and competitive dominance (Kavaliers et al. 2005) modulate the efficiency of fear transfer through observation. However, the specifics of how the social relationship between rats contributes to social fear learning have not been examined.

Previously, we found that fear to a discrete cue could be socially transmitted in rats simply through interacting with a fearful conspecific in the presence of an otherwise benign stimulus (Bruchey et al. 2010; Jones et al. 2014). We demonstrated that some rats displayed conditioned responding to a cue after interacting with a cage-mate during fear memory retrieval (Bruchey et al. 2010). This 3-day “fear conditioning by proxy” (FCbP) paradigm makes use of rats housed in triads. Each rat is assigned to one of three behavioral conditions, resulting in direct fear conditioning (FC), social fear conditioning (fear conditioning by proxy or FCbP), or no fear conditioning (No FC). This paradigm is unique for studying social fear learning because (1) rats freely interact with each other during the social learning session and (2) behavior can be observed both as a pair, during training on day 2, and alone, during the follow-up test on day 3. Testing in the absence of the demonstrator is essential to determine whether learning has occurred by ruling out any motivational or social facilitation effects that can occur when animals are present in the same chamber.

In addition to freezing responses to a conditioned stimulus, rats can emit vocal sounds including sonic calls that are audible to humans as well as most other predators and two subtypes of calls in the ultrasonic frequency range (>20 kHz) (Sales and Pye 1974). Vocalizations in the lower spectrum of the ultrasonic range (around 22 kHz) are typically associated with negative affect (Kaltwasser 1991) elicited in situations where the rodent is fearful, including proximity of a predator (Blanchard et al. 1991). Higher frequency vocalizations (in the 50 kHz range) are affiliated with activities tied to more positive affect (Burgdorf et al. 2000; Knutson et al. 2002; Wöhr and Schwarting 2007).

Social transmission of fear paradigms, including observational fear conditioning, indicate that social fear learning recruits physiological mechanisms that overlap with those engaged during direct fear conditioning [e.g., the amygdala (Jeon et al. 2010; Knapska et al. 2006); activation of the hypothalamic–pituitary–adrenal (HPA) axis and glucocorticoid release (Kavaliers et al. 2003)]. However, the modest effect on behavior (Atsak et al. 2011; Bruchey et al. 2010; Jones et al. 2014; Pereira et al. 2012) leads to questions about other mechanisms behind social fear learning including possible individual differences in stress responding between rats. Glucocorticoids modulate both observation of fear behavior to natural predators (Kavaliers et al. 2003) as well as the formation and maintenance of the social dominance hierarchy (Cordero and Sandi 2007; Timmer and Sandi 2010) and variations in corticosterone levels could contribute to differences in a socially acquired fear response.

Given the similar behavioral output and potential overlap in ethological function, we hypothesize a great degree of similarity in the neural and hormonal mechanisms involved in social fear learning and direct associative fear learning with the main pathways likely preserved between paradigms (Jeon et al. 2010; Knapska et al. 2006; Olsson and Phelps 2007). However, discerning any neural pathways that uniquely subserve fear learning through vicarious experience can determine potential applications of this paradigm beyond traditional fear learning.

Despite extensive knowledge of the etiology of play behavior, intra-specific aggression, social interactions, and social recognition in laboratory rodents (Ferguson et al. 2001; Grant and Mackintosh 1963; Meaney and Stewart 1981; Panksepp 1981; Pellis and Pellis 1987; Pellis et al. 1992; Popik et al. 1992; Thor and Holloway 1982; Vanderschuren et al. 1997), these relationships are generally ignored or treated as nuisance variables in studies of learning and memory. Here, we explicitly tested the impact of intra-cage dominance relationships (determined from observations of social interactions) and ultrasonic vocalizations on fear conditioning by proxy. Next, we disambiguated neural networks that selectively contribute to social transmission of fear and tested the possible role of corticosterone as a predictor of fear behavior in response to a fearful conspecific.

## Methods

### General overview of methods

Four experiments were conducted, each exploring aspects of social fear learning in rats through the fear conditioning by proxy paradigm. On day 1, one rat of each triad was fear conditioned to a tone (80 dB, 5 kHz, 20 s) coterminating with a foot-shock (0.7 mA, 500 ms). On day 2, the fear-conditioned rat (FC rat) was returned to the fear-conditioning chamber accompanied by a cage-mate (FCbP rat) and the tone was played in the absence of the foot-shock. The third rat (No FC rat) remained in the home cage and on day 2 was allowed to freely interact with the fear-conditioned (FC) and fear-conditioned by-proxy (FCbP) rat when they were returned after the fear conditioning by-proxy session on day 2. The following day (day 3), all rats (FC, FCbP, and No FC) were placed in the chambers alone and tested for fear expression (freezing) to the tone.

**Fig. 1:**
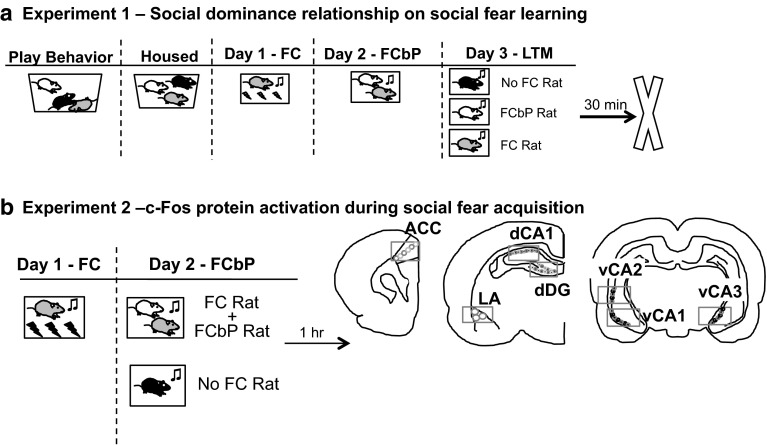
Experiments 1 and 2 design. **a** Experiment 1: Play behavior within a triad was observed 3 weeks prior to fear conditioning by proxy paradigm and used to determine dominance status. One rat of the triad was fear conditioned directly on day 1 (FC). On day 2, the FC rat and a cage-mate (FCbP rat) were exposed to the CS together. On day 3, each rat was exposed to the CS alone as a measure of long-term fear memory. Trunk blood was collected 30 min after LTM test on day 3 for analysis of serum corticosterone levels. **b** Experiment 2 design and counting frames for c-Fos IHC (*circles*). *Rectangle* indicates imaging frame from camera. One rat in the triad was fear conditioned to a tone CS on day 1 (*FC Rat*). The following day, the FC rat and FCbP rat were placed in the chamber together, the third No FC rat was placed in the chamber alone, while the CS was presented and rats were euthanized 1 h after cue presentation. Immunopositive nuclei were counted in the anterior cingulate cortex (*ACC*), the lateral nucleus of the amygdala (*LA*), the CA1 region of the dorsal hippocampus (*dCA1*) and the ventral portions of the CA2, CA3, and CA1 regions of the hippocampus. *Circles* represent the fixed counting frames used for cell quantification, counts were sampled from *circles* outlined in *red* (color figure online)

In experiment 1, the role of social dominance on fear conditioning by proxy was examined by manipulating the fear conditioning group assignment in a triad of rats after observing and classifying rats according to social behaviors within a cage. Freezing, ultrasonic vocalizations (USVs), and serum corticosterone levels were used as endpoints in determining response to social and direct fear learning and social behaviors were incorporated into these analyses (see Fig. 1a for experimental design). For analysis of behavior, rats were divided into groups based on the dominance status of both the FC rat and the FCbP rat (see Table S1 for possible group combinations; Table 1 for acronyms used).

**Table 1:** Acronyms and respective definitions

Abbr.	Full name	Description/example
CS	Conditioned stimulus	Inherently neutral prior to conditioning: tone
US	Unconditioned stimulus	Inherently aversive: foot-shock
FC	Fear conditioning	Direct learning session, or rat designated as individual learner/demonstrator
FCbP	Fear conditioning by proxy	Social learning session, or rat designated as social learner/observer
No FC	No fear conditioning	Rat with no direct experience but housed with rats with direct and social fear experience
LTM	Long-term memory	Memory retention test 24 or 48 h after acquisition; 3 tones played in the absence of the foot-shock
USV	Ultrasonic vocalization	Vocalizations signaling either negative-affect (22 kHz range) or positive-affect (50 kHz range)
D	Dominant	Rat that is the target of most nape contacts
S1	Subordinate 1	Rat that is the preferred target of dominant
S2	Subordinate 2	Rat that is mostly avoidant of dominant
ACC	Anterior cingulate cortex	Anterior portion of the cingulate cortex, important for information processing and cognitive control
LA	Lateral amygdala	Lateral portion of the basolateral complex, important for convergence of fear associations
dHPC	Dorsal hippocampus	Dorsal region of hippocampal formation
vHPC	Ventral hippocampus	Ventral region of hippocampal formation
CA1, CA2, CA3	*Cornu Ammonis* 1,2,3 (latin for Ammon’s horn)	Regions of Ammon’s horn portion of the hippocampal formation: CA1, CA2, CA3 are important for learning and memory
DG	Dentate gyrus	Dentate gyrus portion of hippocampal formation, important for learning

Commonly used abbreviations defined and a brief description or example for each term

**Fig. 2:**
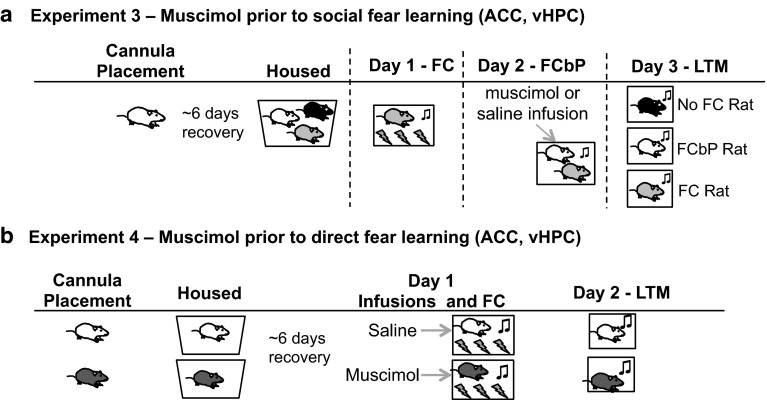
Experiments 3 and 4 design. **a** Experiment 3: Experimental design for muscimol infusions into vHPC or ACC. Rats designated as FCbP (S2 rats) were surgically implanted with bilateral cannula aimed at either the ventral hippocampus or the anterior cingulate cortex and allowed to recover. Twenty minutes prior to fear conditioning by proxy with the FC rat, FCbP rats were infused with either saline or muscimol. The following day, in the absence of the drug, all rats were tested for freezing to the cues. **b** Experiment 4: experimental design. Rats were surgically implanted with bilateral guide cannulae aimed at either the vHPC or ACC and allowed to recover. Muscimol or saline was infused prior to direct CS–US pairings, and rats were tested for freezing to the CS the following day

To investigate the neural mechanisms recruited during the fear conditioning by proxy paradigm, in experiment 2, c-Fos immunohistochemistry was performed using the optimal fear conditioning by proxy conditions determined in experiment 1 (see Fig. 1b for experimental design). Quantifying c-Fos in regions involved in fear learning and social behaviors [e.g., lateral amygdala (LA), anterior cingulate cortex (ACC), dorsal hippocampus (dHPC), ventral hippocampus (vHPC)] allowed us to examine relative neural activity in these regions between each group of rats (FC, FCbP, No FC) at the cellular level. The results of the immunohistochemistry guided the target regions of interest for muscimol micro-infusions in experiments 3 and 4. Muscimol acts as a temporary inactivator of the region by enhancing GABA inhibition, allowing experimenters to disentangle obligatory from permissive roles of the relevant brain regions involved in acquisition of fear conditioning by proxy (experiment 3; see Fig. 2a for experimental design) and direct fear conditioning (experiment 4; see Fig. 2b for experimental design). Rats were surgically implanted with bilateral guide cannulae aimed at the regions of interest (ventral hippocampus or anterior cingulate cortex) 1 week prior to microinfusions of either muscimol or saline before acquiring fear through direct (experiment 4) or indirect (e.g., social; experiment 3) experience.

### Subjects

#### Experiment 1

Subjects were male Sprague–Dawley rats weighing approximately 400–500 g at time of behavioral test. All animals were bred at the University of Texas at Austin using males (275–300 g at arrival) acquired from Harlan (Houston, TX, USA) and females (225–275 g at arrival) acquired from Charles River (Wilmington, MA, USA) in an attempt to diversify genetic lines. Breeder adult males were removed before the birth of the litter and pups were weaned into same sex triads with littermates at post-natal day 21 (p 21). The male pups were allowed to mature with minimal disturbances and limited to routine animal husbandry, and the female pups were used for a separate fear conditioning by proxy experiment (see Jones et al. 2014). No group had more than one cage of rats from a single litter, and 279 total animals were used. Rats were housed in clear plastic cages and maintained on 12-h light/dark cycle with lights on at 0700 h. Standard rat chow and water were provided ad libitum. Procedures were conducted in compliance with the National Institutes of Health Guide for the Care and Use of Experimental Animals and were approved by The University of Texas at Austin Animal Care and Use Committee.

Six cohorts of animals were run resulting in some variation in age at both dominance test (mean = 96 days; max = 109 days; min = 73 days; SD = 11 days) and behavioral test (mean = 116 days; max = 139 days; min = 100 days; SD = 12 days) as well as amount of data collected for each cohort. Each cohort included animals from all groups.

#### Experiment 2

Subjects were identical to the subjects bred for experiment 1. In total, 36 male rats were used for immunohistochemistry in experiment 2.

Each triad consisted of one rat to be fear conditioned (FC), one rat to be fear conditioned by proxy (FCbP), and one rat that would not be conditioned (No FC control). Rats were euthanized 1 h after 3 CS exposures on day 2 in order to investigate neural activity after the fear conditioning by proxy paradigm. Groups were assigned in such a way that the FC rat was the dominant rat and the FCbP rat was the subordinate 2 (S2).

#### Experiment 3

Subjects and groups were identical to experiment 2. Ninety-nine animals were used in experiment 3.

#### Experiment 4

Subjects were male Sprague–Dawley rats 325–350 g ordered from Harlan (Houston, TX). Rats were single housed prior to surgery and remained housed individually and therefore did not require onsite breeding and weaning into sibling cages. All rats underwent direct fear conditioning. Twenty-three rats were used in experiment 4.

### Apparatus and stimuli (experiments 1–4)

All fear conditioning procedures took place in standard conditioning chambers (30.5 cm W × 25.4 cm D × 30.5 cm H) equipped with metal walls and stainless-steel rod floors connected to a shock generator (Coulbourn Instruments, Allentown, PA). Chambers were enclosed in acoustic isolation boxes (Coulbourn Instruments) and lit with a red light. Behavior was recorded with closed circuit cameras (Panasonic™ WV-BP334) mounted on the top of each unit, and these videos were watched manually to quantify behaviors of interest (freezing and social contact, described below). The chambers were wiped with soap and water between each session. Stimulus delivery was controlled using Freeze Frame software (Coulbourn Instruments). The conditioned stimulus (CS) was a tone (5 kHz, 80 dB) 20 s in duration, and the unconditioned stimulus (US) was a 0.7 mA foot-shock 500 ms in duration.

### Procedures

#### Fear conditioning by proxy design (experiments 1–3)

Each rat in a caged triad was assigned to one of the following behavioral conditions according to dominance rank: FC, FCbP, or No FC. All tests were performed during the light portion of the light cycle between 1400 and 1600 hours.

Fear Conditioning (FC; Day 1)

On the fear-conditioning day, after a 10-min habituation period, one rat per triad (FC Rat) received three presentations of the CS (variable inter-trial interval (ITI), mean = 180 s, range 120–240 s), each co-terminating with the US. After fear conditioning, all rats were returned to their home cages.

Fear Conditioning by Proxy (FCbP; Day 2)

One day after conditioning, the fear-conditioned rat was returned to the chamber accompanied by a previously naïve cage-mate, with no prior exposure to CS or US (FCbP Rat). The rats were allowed to interact with each other freely while the CS was presented three times (variable ITI, mean = 180 s). The third rat of the triad (No FC) remained in the home cage.

Long-term Memory Test (LTM; Day 3)

Twenty-four hours after fear conditioning by proxy, each rat (FC, FCbP, and No FC) was placed in the chamber alone and received a long-term memory test (3 CS presentations, variable ITI, mean = 180 s) to assess fear expression to the CS.

#### Social interaction tests for dominance (experiments 1–3)

Dominance within each triad of males raised together was assessed at approximately p 90 and used for group assignment, 2 weeks prior to adult behavioral testing (fear conditioning by proxy) in experiments 1–3. Dominance tests were performed according to the methods of Pellis and Pellis (1991), Pellis et al. (1993) in order to obtain data from freely behaving adult rats. In this paradigm, social interactions were recorded with a digital video camera (Sony™ HandyCam™) in 12-min sessions during the first 4 h of the animal’s dark cycle starting when the animals were approximately 90 days old.

To encourage active engagement of social behaviors, rats were single housed for 24 h (Panksepp and Beatty 1980) in clear cages identical to their home cage and arranged adjacent to previous cage-mates within the animal colony, with middle or end placements randomly determined. After 24 h of single housing, the original three rats from the triad were placed in a clear plastic bin (58.4 cm × 41.3 cm × 31.2 cm) with cedar chips as flooring (similar to, but larger than their home cages) and allowed to freely interact for 12 min under red light illumination.

After 12 min of recording, the triad was housed together as a group for at least 24 h; they were then isolated for another 24 h and a new play session was recorded at the completion of the isolation period. This was repeated until 3 social interaction sessions were recorded. Rats were not housed individually for longer than 24 h at a time and throughout their lives did not spend more than 72 cumulative hours in social isolation. Rats were marked with a black Sharpie™ marker for identification and allowed to habituate to the bin used for play behavior for 10 min 72 h before the first rehousing (prior to any single housing).

#### Determination of social dominance (experiments 1–3)

Social behaviors were recorded and combined for the three sessions, resulting in a 36 min sample of social behavior that occurred over 3 different time points. When rats fight, the attacker animal will attempt to contact part of the target animal usually in an effort to bite the body target. In fights stemming from ritualized aggression, the target is usually the nape and in fights stemming from overt aggression, this target is the rump or flanks (Pellis and Pellis 1987; Siviy and Panksepp 1987). Piloerection and threat posturing not present during play will also accompany serious fights (Adams and Boice 1983; Blanchard et al. 1977; Poole and Fish 1975). Although play fighting is most common in juvenile animals, rats will continue to engage in socially coordinated motor behavior that resembles play fighting past sexual maturity. Piloerection, freezing, and threat postures were not observed in any of the recorded sessions here. Social interaction videos were watched and scored for offensive behaviors (attack: contacts directed toward the nape or other body target) and defensive behaviors in initial response to attack (withdrawal of the nape: evasion or facing defense).

Nape contacts were counted when one rat brought his snout within approximately 2 cm of the nape of another rat. No other body targets were observed in these sessions. If the target animal responded to the nape contact, the response was scored as either: evasion (target animal runs away or pulls nape away from attacker) or any of the following forms of facing defense: counterattack (target animal turns to face attacker and launches an attack of his own; boxing was included in this if it was in response to a nape contact), full rotate to supine (target animal rotates along his longitudinal axis to supine position, blocking access to the nape), or half rotate (target animal rotates laterally to block nape contact but feet remain planted) (see table S2 for description of behaviors counted).

Two trained observers watched all videos in slow motion to distinguish which rat of the triad initiated each contact and characterized the initial defensive response of the target animal to the initiated contact (inter-rater correlation for behavior counts/session/rat: *r* = .97, *P* < .001). Nape contacts and defensive responses were counted for individual rats in each triad during the play behavior tests in order to determine whether the cage possessed a dominant (D), subordinate 1 (S1), and subordinate 2 (S2) rat as described in detail in Pellis and Pellis (1991), Pellis et al. (1993). Briefly, the research conducted by Pellis et al., describes that when adult rats (post-natal day 80) are housed together, the dominant rat is the recipient of a disproportionate share of nape contacts (snout of one rat approaches and makes brief contact with the nape of another) (Pellis et al. 1993). When presented with an unfamiliar intruder rat, the rat that received the most nape contact from other rats in the cage was most likely to initiate aggressive attack against the intruder (a commonly used marker of dominance) (Pellis and Pellis 1991).

Dominance assignments were made for each cage of triads by considering only the play behavior of that specific cage using the criteria below.

*Dominant rat (D)*: When nape contacts were tallied, if one rat of the triad received a disproportionate share of contacts (>33.3 %), this rat was identified as the D rat (actual share of nape contacts was 40.3–65.9 %). The D rat was most likely to respond to nape contacts by turning to face the attacker and launching a successful counterattack that resulted in the initial attacker in a supine position.

*Subordinate 1 rat (S1)*: The S1 rat was identified as the preferred target of nape contacts initiated by the D rat (>50 % of D nape contacts toward S1). Additionally, the S1 rat initiated the most nape contacts and consequently, most of the play that occurred within the cage was between the D and S1 rat.

*Subordinate 2 rat (S2)*: The remaining subordinate rat was the S2 rat and was mostly avoidant.

Three sessions of social interactions were recorded and nape contacts were similar across the three recording sessions. Only cages with a clear dominance hierarchy of D, S1, S2 were continued in the set of experiments presented here (Fig. S1) (see supplementary methods).

This dominance assignment was used to assign groups and determine which rat of the triad was fear conditioned directly (FC), fear conditioned by proxy (FCbP), or exposed to the CS only (No FC). In our first experiment, every possible combination of dominance status (D, S1, S2) and fear conditioning group (FC, FCbP, or No FC) within a triad was run through the fear conditioning by proxy paradigm (see Table s1 for possible group assignments).

#### Competitive dominance

The use of nape contacts to establish a dominance hierarchy was validated in a subset of animals by comparing the monopolization of a desired resource (sweetened milk) within a triad with the dominance assigned through social interactions. Cages were first run through social interaction tests, and a D, S1, S2 hierarchy status was assigned within each cage using the nape contact/defensive response method described above. One week later, a conical ceramic food dish filled with sweetened milk was placed in the recording bins with each triad (see supplemental methods). This food dish was designed so that only one rat could drink out of it at a time. The amount of time that each rat spent drinking was measured and plotted according to play behavior dominance assignment.

#### Ultrasonic vocalization recordings (experiment 1)

WAV files were recorded from the chambers for the entire session on each day using Avisoft-recorder (Avisoft Bioacoustics, Berlin) with a sampling rate of 250 kHz. Clips were imported for analysis to Avisoft SASLab Pro (Version 5.2, Avisoft Bioacoustics). Ultrasonic vocalization (USV) detection was performed on the spectrograms by a trained observer blind to experimental condition. Duration, frequency, and peak amplitudes were measured for each call along with occurrence before, after, or during CS presentation. Calls were categorized according to their frequency range with 18–30 kHz calls (22 kHz calls) indicating negative affect and 40–90 kHz calls (50 kHz frequency range) indicating positive affect. There were equipment malfunctions in USV collection for 2 cohorts. This resulted in approximately 30 animals without any USV data and another 30 animals that did not have data for frequencies above 35 kHz. These animals were included in the behavioral analysis but were not included for analysis of vocalization data that was not obtained.

#### Serum collection (experiment 1)

Rats were euthanized 30 min following long-term memory tests (Day 3) with a lethal dose of sodium pentobarbital (Euthasol) and immediately decapitated. Trunk blood was collected and allowed to clot for approximately 20–30 min at room temperature. Serum was separated via centrifugation (1500*g* for 10 min) and stored at −80 °C until assays. There was an error in blood collection for one of the cohorts resulting in approximately 30 rats that are included in the behavioral analysis but were without serum.

#### Tissue collection (experiment 2)

One hour after fear conditioning by proxy rats were injected with a lethal dose of sodium pentobarbital (Euthasol) and intracardially perfused with 4 % paraformaldehyde. All perfusions took place between approximately 2 and 4 pm to minimize effects of hormonal fluctuations that occur throughout the day. Brains were post-fixed in 4 % paraformaldehyde for 24–48 h and then transferred to 30 % sucrose in phosphate-buffered saline (PBS) for cryoprotection. Coronal sections, 35 μm thick, were taken with a freezing microtome of the entire brain and stored in PBS in 4 series, resulting in every 4th tissue slice undergoing immunohistochemical processing (see supplemental methods for histological methods).

#### Surgical procedures: temporary inactivation before FCbP (experiment 3) or direct FC (experiment 4)

Six days before behavioral testing, rats were single housed and implanted with bilateral guide cannulas (26 gauge, Plastics One) aimed at either the ventral hippocampus (12 mm) or the rostral anterior cingulate cortex (5 mm) (see supplemental methods for surgical procedure). After recovery (6 days), rats were either infused with the GABAa agonist muscimol or saline just prior to either social acquisition of fear (fear conditioning by proxy; Fig. 2a for experimental design) or direct acquisition of fear (direct fear conditioning; Fig. 2b for experimental design) (see supplemental methods for infusion methods).

#### Direct or social fear acquisition after muscimol infusion

After infusion of either saline or muscimol was complete (see supplemental methods), rats were returned to their home cages and returned to the colony for 20 min before being transported to the fear conditioning chambers. They were then either fear conditioned directly as described in Day 1 (experiment 4) or they were paired with a previously fear-conditioned cage mate as described in Day 2 (experiment 3). Twenty-four hours later, rats were tested for long-term memory to the CS in the absence of the drug with 3 non-reinforced CS presentations.

### Data scoring and analysis

#### Freezing

Video files recorded from each day of the fear conditioning by proxy paradigm were watched by a trained observer blind to group assignment to quantify the amount of time the rat(s) froze during each CS presentation. Freezing was defined as the absence of any movement, excluding breathing and whisker twitching. The total number of seconds spent freezing throughout the CS presentation is expressed as a percentage of CS duration (20 s). Freezing was analyzed for each day of the fear conditioning by proxy paradigm. A randomly chosen sample consisting of approximately 40 % of the videos were scored by a second blind observer and inter-rater reliability was high (*r* = .98, *P* < .001).

#### Social contact during fear conditioning by proxy

Social contact was defined as any physical contact or interaction excluding accidental contact made in passing. This contact was measured as the percentage of time that the FCbP rat spent engaging in social contact with the fear-conditioned (FC) rat throughout either the duration of each CS or during the immediate 20 s following the termination of each CS on day 2 of the fear conditioning by proxy paradigm. This contact included any of the following behavior types: allogrooming, paw contact, body contact, sniffing, nose to-nose contact, and play (defined in Bruchey et al. 2010) and was scored by an observer blind to group assignment through video observation. As with freezing, a randomly chosen sample of videos were scored by a second observer blind to group assignment and inter-rater reliability was high (*r* = .96, *P* < .001).

#### Ultrasonic vocalization analysis (experiment 1)

Ultrasonic vocalizations were recorded during fear conditioning on day 1, fear conditioning by proxy on day 2, and long-term memory on day 3. Vocalizations were analyzed blind to group assignment over the entire behavioral session without regard for CS presentation (see supplemental methods).

#### Corticosterone analysis (experiment 1)

Concentration of serum hormones were measured using Enzyme-linked Immunosorbent Assays (ELISA) kits for corticosterone (Cayman Chemicals) according to the manufacturer’s protocol. All samples were run in duplicate at two dilutions (1:400 and 1:800) in order to ensure concentrations that fell within the linear range of the standard provided with each kit (see supplemental methods). Ten corticosterone plates were run in order to obtain values for all 116 samples (see supplemental methods).

#### Cell quantification and analysis (experiment 2)

Immunopositive nuclei were counted by an observer blind to experimental group using a fixed counting frame in a given structure as described for each specific experiment. The density of c-Fos positive cells was calculated by dividing the sum of cells counted within a set counting frame for each area by the total area of the counting frames. When possible, six sections were taken from each brain for imaging and an average c-Fos count was calculated for each rat per region of interest using fixed counting frames (Fig. 1b; see supplemental methods).

### Statistical analysis

Statistical analyses were performed with IBM SPSS Statistics version 22.0 for Mac. Unless otherwise noted, group differences for a dependent variable of interest were determined using ANOVA with fear conditioning group (FC, FCbP, or No FC), dominance assignment (D, S1, or S2) as the between subject factors and significant main effects were followed up with post hoc Tukey mean comparisons. Results were considered significant at *P* < .05. When applicable, a priori planned comparisons were performed with two-tailed independent samples *t* tests and these tests are specifically noted.

Bivariate correlations were used to examine the relationship between a priori determined outcome variables and possible predictors. The Pearson product-moment correlation was used unless underlying assumptions were not met. When parametric assumptions were not met, the Spearman correlation coefficient for ranked data was instead used for analysis.

In the current set of experiments, the recordings of social interactions used to assess dominance status provided offensive and defensive information reflective of the social relationship within the triad of rats and allowed for more in depth analysis of how pre-existing social relationships, and the social behaviors that accompany them, combine with social behavior exhibited during behavioral tests to predict the freezing response displayed after social fear learning. Exploratory relationships between variables were analyzed using linear regression with pre-determined predictor variables entered into the model. Hierarchical multiple regression analyses were used to explore the contribution of social behaviors between FC and FCbP rat and the contact that occurred during social learning on day 2, to freezing displayed on day 3 by FCbP rats. This method of hierarchical analysis was chosen because although we have previously demonstrated that social contact on day 2 significantly predicts freezing in the FCbP rat on day 3, it remains unknown how much of this variance in freezing is accounted for by social contact or shared by general indicators of the social relationship between the two rats (e.g., play initiation and response type). Additionally, with this analysis, we can determine the unique contribution that each social behavior has on freezing using continuous variables instead of grouping play into finite categories.

Social behaviors were blocked into two different levels organized in the hierarchy by when they occurred: (1) likelihood of play behavior between the FC and FCbP rat during dominance tests and (2) social contact during the FCbP session on day 2. Factors analyzed in the first level included: percent of nape contacts within a cage that were initiated by the FCbP rat toward the FC rat (FCbP Nape FC) and vice versa (FC Nape FCbP) as well as the likelihood that these nape contacts elicited any of the three main defensive responses measured for dominance assignment (evasion, counter, and rotate (only full rotate to supine was used)) including an indicator of which rat responded with which behavior (e.g., FC Evade FCbP is the percentage of nape contacts initiated by the FCbP rat toward the FC rat that resulted in evasion by the FC rat across all three play behavior recordings). Both overall nape contact and response to nape approach are used to determine dominance status and consequently hypothesized to potentially predict the proficiency of the social transmission of fear in the FCbP paradigm. It is important to point out that the behaviors included in the first level of analysis occurred approximately 2–3 weeks prior to fear conditioning by proxy and recording of the dependent variable measured here (FCbP Freezing on day 3). The second level of the hierarchical regression analysis consisted of two possible contributing factors: social contact between the FC and FCbP rat during the cues of the fear conditioning by proxy paradigm and social contact immediately post cue during the fear conditioning by proxy session. This method of hierarchical regression analysis allowed for analysis of the relationship between social contact and long-term memory (LTM) freezing independent of the pre-existing social behaviors that occurred between rats.

## Results

### Dominance hierarchy within a cage can be determined by intra-cage social behaviors

Using observations of social interactions, dominant status (D) was assigned to rats that received the majority of nape contacts within the cage and responded to nape contacts with significantly more counter attacks than subordinates (*F*(2,116) = 8.6, *P* < .001). Subordinate rats responded to nape contacts from the dominant by rotating onto their backs significantly more than dominants did in response to subordinates (*F*(2,116) = 8.22, *P* < .001). Subordinates were further divided based on nape contacts initiated by the dominant rats with subordinate 1 rats (S1) receiving more nape contacts than the subordinate 2 (S2).

The social interaction dominance assignment was compared to competitive dominance in a subset of rats (9 cages, *n* = 27) by first determining dominance from nape contacts and then putting the same rats in competition for a desired resource (sweetened milk). One of the cages did not engage in any play behavior during any of the 3 recorded play sessions and was not included in analysis. For the remaining 8 cages, the D rats, as indicated from nape contacts, spent significantly more time drinking across the entire 7-min recording session than either the S1 (post hoc Tukey *P* < .01) or the S2 rat (post hoc Tukey *P* < .001; repeated measures ANOVA between group effect *F*(2,21) = 12.32, *P* < .001) (Fig S2).

### Social dominance predicts fear transmission “by proxy”

Learned fear to the CS was assessed by measuring freezing displayed during CS presentation on each day of the fear conditioning by proxy paradigm. Despite increased freezing in the D rat during the final cue of fear conditioning with direct CS–US pairings on day 1 (one-way ANOVA, *F*(2,57) = 3.55, *P* = .035) (Fig. S3a), follow-up retention tests on subsequent days revealed no differences in freezing among dominance assignments after direct fear conditioning (Fig. S3b). This suggests that although the D rat may respond moderately more while a threat is immediately present, there are no differences in actual retention of the fear during retrieval at a later time.

**Fig. 3:**
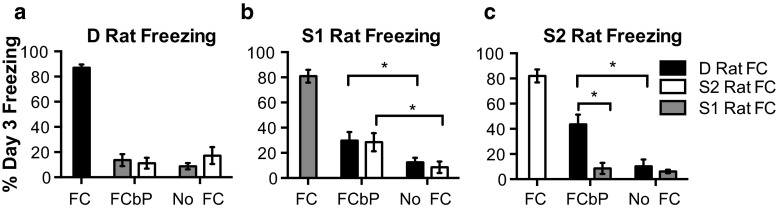
Subordinate FCbP rats froze during CS presentation on day 3 when paired with higher ranked fear expressing cage-mates. **a**–**c** Freezing on day 3 in each dominance subtype. There was no effect of dominance status on freezing after direct FC (*far left bars of each panel*). **a** The D FCbP rats did not freeze significantly more to the cues than D No FC rats after fear conditioning by proxy with either S1 (*blue bars*, *n* = 12) or S2 (*white bars*, *n* = 11) (both *P*s > .05). **b** S1 FCbP rats froze significantly more after fear conditioning by proxy with either a FC D rat (*black bar*; *n* = 10) or S2 rat (*white bar*; *n* = 10) than S1 rats that were not fear conditioned (*P*s < .05) but C) S2 rats only showed socially acquired freezing after fear conditioning by proxy with a FC D rat (*black bar*; *n* = 9) (*P* < .05). *Error bars* ± SEM (color figure online)

The dominance relationship between the observer (FCbP) and demonstrator (FC) rat was crucial in determining the amount of fear that was transmitted socially (dominance of FC rat × dominance of FCbP rat interaction, *F*(1,54) = 6.07, *P* = .02). When each rat was tested alone on day 3 (Fig. 3), subordinate rats displayed more freezing after a social learning session with a dominant demonstrator. S1 rats acquired fear by proxy after observing and interacting with either D or S2 rats on day 2 (Fig. 3b) and S2 rats only froze after fear conditioning by proxy with a D rat (Fig. 3c). D rats did not learn to fear a cue socially from either subordinate (Fig. 3a).

**Fig. 4:**
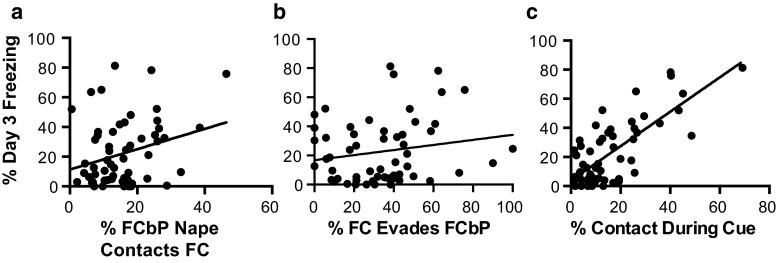
Social behaviors as predictors of socially acquired fear. **a**–**c** Relationships between social behaviors and day 3 freezing in FCbP rat. **a** Nape contacts initiated by the FCbP rat toward the FC rat as a percentage of total nape contacts and **b** likelihood of evasion when FCbP nape contacts FC rat (percent of nape contacts that resulted in evasive response of FC rat) during the play behavior session were entered in the first step and contributed a small but significant amount. **c** Social contact during the cues of the fear conditioning by proxy session accounted for the largest amount of unique variance in LTM freezing displayed by the FCbP rat the following day

Consistent with our previous applications of the fear conditioning by proxy paradigm indicating that social contact that occurs between the FC and FCbP rat during the CS in male rats (Bruchey et al. 2010) and after the CS in female rats (Jones et al. 2014) is positively correlated with LTM freezing displayed by the FCbP rat, it was hypothesized that social contact that occurred during the FCbP cues would account for a significant amount of variability in freezing displayed at LTM between the fear conditioned by proxy rats. Here, the behaviors observed in the dominance tests allowed us to determine which social behaviors were essential for predicting later social fear learning. In line with our earlier studies (Bruchey et al. 2010; Jones et al. 2014), we found that social contact that occurred during the cues of fear conditioning by proxy remained the strongest unique predictor of LTM freezing (see table s3 for regression model; semi-partial correlation = .34, *P* < .01) (Fig. 4c). Overall play initiation by the FCbP rat toward the FC rat (semi-partial correlation = .2; *P* = .02) and high evasion of FC rat in response to social engagement attempts from the FCbP rat (semi-partial correlation = .18; *P* = .04); both indicators of social asymmetry between observer–demonstrator pairs uniquely contributed a moderate, but significant amount (Fig. 4a,b).

### When present, negative-affect ultrasonic vocalizations (22 kHz) contribute to social fear transmission

**Fig. 5:**
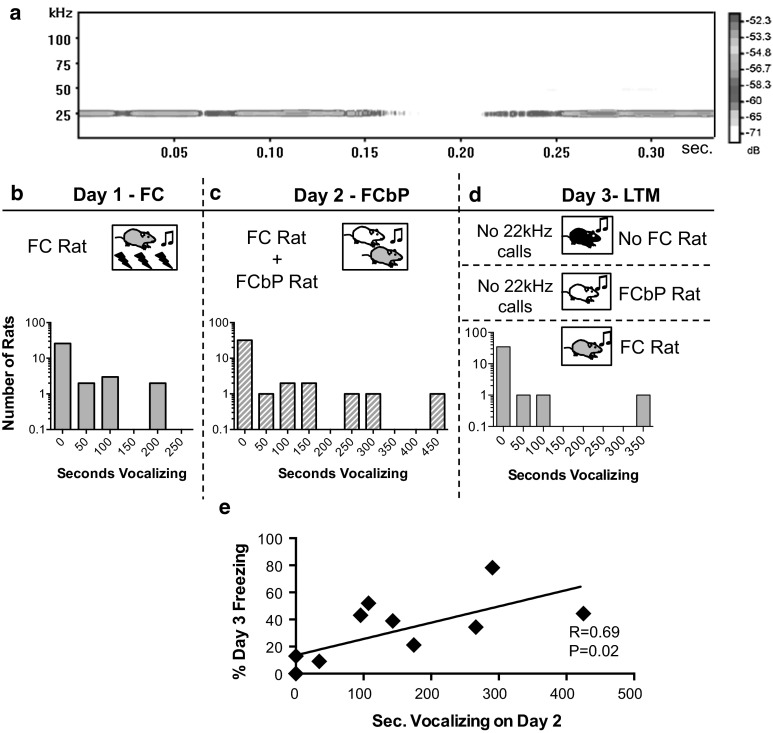
Infrequent negative-affect ultrasonic vocalizations correlate with socially transmitted fear. **a** Sample spectrogram of a 22 kHz vocalization. **b**–**d** Frequency histograms of the number of subjects that elicited negative-affect vocalizations in the 22 kHz range, represented graphically on a logarithmic scale, during **b** fear conditioning on day 1, **c** fear conditioning by proxy on day 2, and **d** long-term memory tests on day 3 indicate that most rats do not vocalize in the 22 kHz range at all and only directly fear-conditioned rats vocalize on day 3. **e** Of the rats that do vocalize (*n* = 10), the total duration of 22 kHz calls during the FCbP session was positively correlated with freezing displayed by the FCbP rat the following day (*P* < .05)

In contrast with previous research indicating that 22 kHz vocalizations (Fig. 5a) are essential for the social transmission of fear in rats (Kim et al. 2010), we found that in the fear conditioning by proxy paradigm, the majority of rats did not vocalize at all during the social transmission of fear on day 2 (Fig. 5c), and this did not preclude a conspecific from learning about associative fear. However, in line with previous work (Atsak et al. 2011; Kim et al. 2010), we found that of the rats that did vocalize in the 22 kHz range during fear conditioning by proxy on day 2 (*n* = 10), the duration of those vocalizations was positively correlated with the freezing displayed by the observer the following day (Fig. 5e) (*R*(10) = .69, *P* = .02).

Unfortunately, the design of the fear conditioning by proxy paradigm did not allow us to distinguish which rats were vocalizing during the FCbP session on day 2, when two rats were in the chambers simultaneously. Any data collected on this day could be either from the FC rat only, the FCbP rat only, or a combination of the two. From the frequency histograms (Fig. 5d), only FC rats vocalized in the 22 kHz frequency range during LTM tests on day 3. It seems probable, then, that the 22 kHz vocalizations observed on day 2 were most likely emitted from the FC rat although we cannot rule out the possibility that these vocalizations, or a subset thereof, came from the FCbP rat.

Further investigations into what may cause some rats to vocalize during this paradigm when others remain silent revealed that the specific relationship between the two rats in the chamber, as determined by observations of play behavior at an earlier time point, predicted the amount of calling (regression model *F*(10,27) = 2.655, *P* = 0.021). Specifically, nape contacts initiated by the FC rat toward the FCbP rat (*β* = 0.406, *P* = 0.037) and reduced likelihood of countering in response to the FC rat (*β* = −0.434, *P* = 0.029) (see Table s4 for full regression model) both contributed to the amount of 22 kHz vocalizations on day 2.

Across all three days, there was a higher occurrence of ultrasonic vocalizations in the 50 kHz frequency range and the number of calls emitted was not normally distributed, with a small portion of rats emitting nearly constant 50 kHz vocalizations (Fig. S4b, c, d). The total number of 50 kHz calls that occurred during the social learning session on day 2 was negatively correlated with freezing displayed by the FCbP rat the following day (Spearman’s rho *r*_s_ = −.46, *P* = .016, *n* = 27) (Fig S4e).

### Dominance status and fear behavior interact to influence corticosterone response to cues

**Fig. 6:**
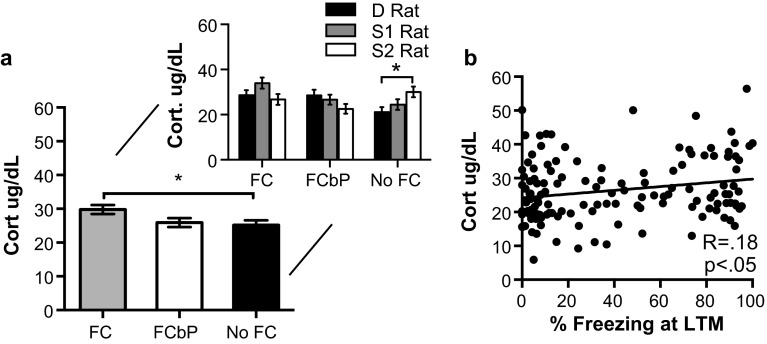
Serum corticosterone on Day 3 correlates with freezing on day 3 and dominance status. **a** FC rats (*n* = 39) had significantly higher corticosterone values than No FC rats (*n* = 37). When these values were further divided based on dominance assignment (*inset*), No FC S2 rats (*n* = 12) had significantly increased corticosterone than No FC D rats (*n* = 12). **b** Freezing on day 3 was moderately but significantly correlated with serum corticosterone levels measured 30 min after LTM test (*n* = 116)

Appropriate behavioral responses to threatening stimuli and stressful situations rely on activation of the hypothalamic–pituitary–adrenal (HPA) axis to shift the neuroendocrine response to stress. Here, we used enzyme-linked immunoabsorbent assays (ELISAs) to measure circulating levels of the end-product of the HPA axis, corticosterone. Rats that were fear conditioned directly had higher levels of serum corticosterone on day 3 than rats that were not fear conditioned (two-way ANOVA effect of FC group: *F*(2,124) = 3.328, *P* = .039; post hoc Tukey: *P* = .044) (Fig. 6a). Although there was no significant effect of dominance on circulating levels of corticosterone overall (two-way ANOVA effect of dominance: *F*(2,124) = .813, *P* = 0.446), there was a significant dominance status × fear conditioning group interaction (*F*(4,124) = 3.591, *P* = .008) which was followed up by analyzing the effect of dominance on each fear conditioning group individually. Consistent with multiple lines of research on subordination stress (Blanchard et al. 1993), No FC rats that were the subordinate 2 had significantly higher levels of corticosterone than No FC dominant rats (one-way ANOVA *F*(2,41) = 4.131, *P* = .023; post hoc Tukey *P* = .018) (Fig. 6a inset). Regardless of FC group and dominance assignment, corticosterone levels were significantly positively correlated with freezing at LTM (Pearson *R(*132) = .175, *P* = .045) (Fig. 6b).

### Neural pathways of FCbP overlap with those for direct FC, but also selectively require anterior cingulate cortex

**Fig. 7:**
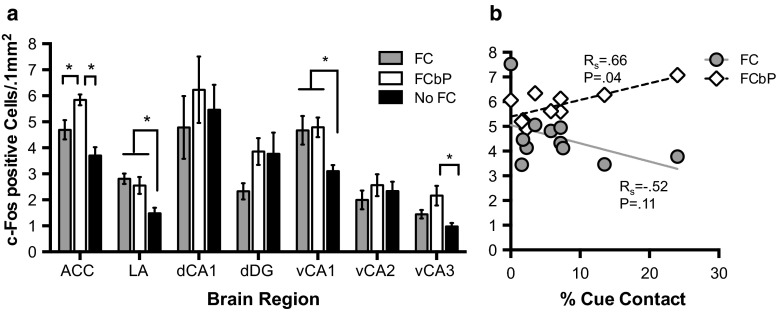
Increased c-Fos activity in ACC and vCA3 on day 2 in FCbP rats. **a** The ACC and vCA3 were uniquely activated in FCbP acquisition. Retrieval of a direct FC memory and acquisition of FCbP both activated the LA and vCA1. *Error bars* ± SEM. **b** Social contact on day 2 predicted c-Fos activity in the ACC in FCbP rats (*n* = 10) but not FC rats (*n* = 11)

The neural processes underlying fear conditioning by proxy were evaluated in parallel with those involved in direct fear conditioning using immunohistochemistry to map transcription of the immediate early gene *c*-*fos* as a surrogate marker of neuronal activity (Greenberg et al. 1986; Sagar et al. 1988). Given the nature of the paradigm, in order to compare animals within a session, activation of a region after social fear acquisition (FCbP Rat) was compared to activity after either retrieval of a directly fear-conditioned memory (FC Rat) or simply presenting the CS to an animal with no previous CS association (No FC rat). For each region examined, c-Fos activity was compared between FC groups and the statistical values are presented in Table 2. We found that both social acquisition and retrieval of directly conditioned fear activated the lateral amygdala (Fig. 7a), a region with a well-documented role in the convergence of associative fear information (LeDoux 1992; LeDoux et al. 1990; Maren 2001) and the ventral CA1 region of the hippocampus (Fig. 7a). We additionally found that acquisition of fear conditioning by proxy uniquely activated neurons in the anterior cingulate cortex (ACC) and ventral CA3 region of the hippocampus at relatively increased levels compared to rats that were retrieving a direct fear memory or rats that had no previous fear experience (Fig. 7a). However, only activity in the CG1 portion of the ACC corresponded with behavior in the FCbP rat (e.g., social contact during the cues) (Spearman’s rho *r*_*s*_ = .66, *P* = .039, *n* = 10) (Fig. 7b). The fear-conditioned rats did not show the same relationship of c-Fos and social contact (ACC *r*_*s*_ = −.515, *P* = .11, *n* = 11) (Fig. 7b), which leads us to conclude that it is not the occurrence of contact, *per se*, that activates this region, but likely a process exclusive to the acquisition of fear information “by proxy”.

**Table 2:** Statistical analyses of c-Fos activity

Brain region	ANOVA	Post hoc Tukey mean comparisons		
		FC versus FCbP	FC versus No FC	FCbP versus No FC
ACC	*F*(2,27) = 7.97, *P* = .002	**	–	**
LA	*F*(2,33) = 8.28, *P* = .001	–	**	**
dCA1	*F*(2,23) = .398, *P* = .676	–	–	–
dDG	*F*(2,22) = 2.814, *P* = .084	–	–	–
vCA1	*F*(2,26) = 4.40, *P* = .023	–	*	*
vCA2	*F*(2,16) = .54, *P* = .595	–	–	–
vCA3	*F*(2,26) = 4.69, *P* = .018	–	–	*

One-way ANOVAs were followed up with Tukey mean comparisons to compare c-Fos activity within each region of interest

*ACC* anterior cingulate cortex, *LA* lateral amygdala, *dCA1* dorsal CA1 region of hippocampus, *dDG* dorsal dentate gyrus; *vCA1* ventral CA1 region of hippocampus, *vCA2* ventral CA2 region of hippocampus, *vCA3* ventral CA3 region of hippocampus

* *P* < .05

** *P* < .01, *P* > .05

**Fig. 8:**
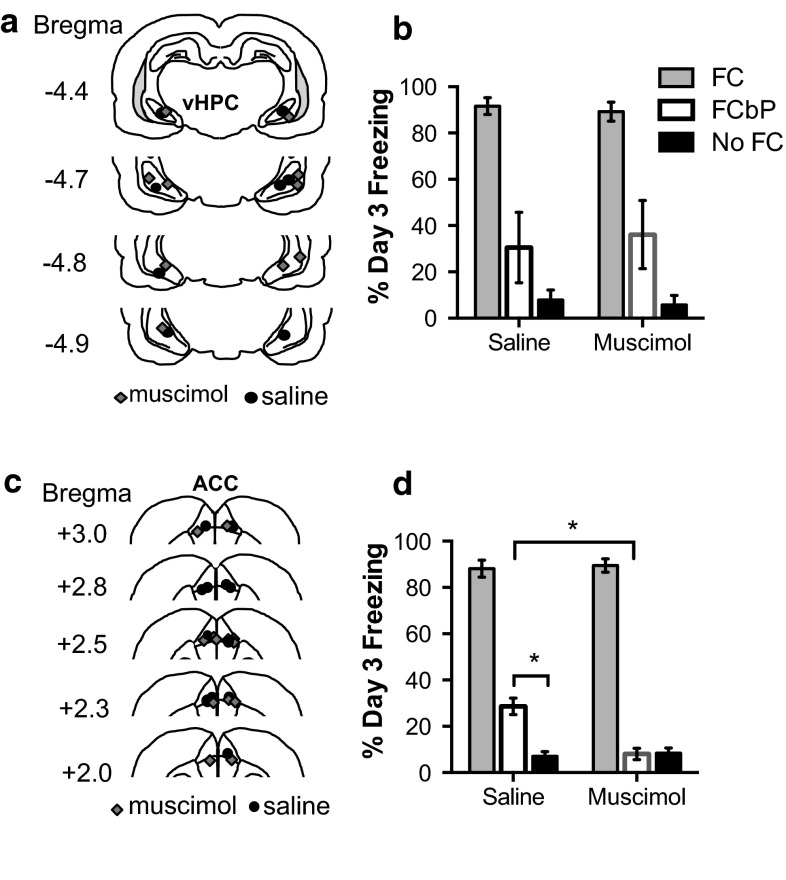
Temporary inactivation of ACC but not vHPC prior to FCbP prevents social fear transmission. **a** Cannula placement in the ventral hippocampus, **b** temporary inactivation of the vHPC did not influence freezing on day 3 after FCbP (saline *n* = 4; muscimol *n* = 5). **c** Cannula placement in ACC. **d** Freezing on day 3 LTM tests indicates that inactivation of the ACC prevented fear acquisition through the FCbP paradigm (saline *n* = 10; muscimol *n* = 8)

Following these results, we next temporarily inhibited activity in the vHPC and ACC by increasing GABAergic transmission with intracranial micro-infusions of the GABA_A_ receptor agonist, muscimol, prior to either the social acquisition or direct acquisition of fear. We found that the anterior cingulate cortex (Fig. 8c), but not the ventral hippocampus (Fig. 8a), is necessary to evaluate the fear conditioning by proxy session in order to express a freezing response when tested the following day (Fig. 8b, d) (FCbP rat day 3 freezing vHPC: *t*(7) = .26, *P* = .9, ACC: *t*(13.9) = 3.17, *P* = .001).

**Fig. 9:**
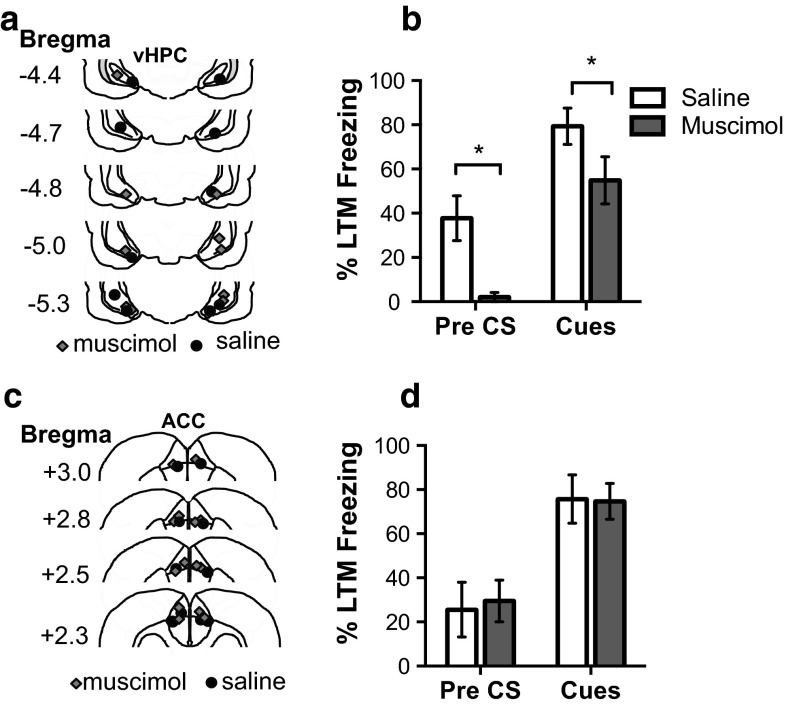
Temporary inactivation of vHPC but not ACC prior to direct FC reduces freezing during LTM tests. **a** Locations of cannula tips in ventral hippocampus. **b** Freezing during long-term memory tests 24 h after infusion of either muscimol (*n* = 5) or saline (*n* = 5). Rats infused with muscimol into the vHPC froze less than saline rats to both the cues and in the 20 s immediately preceding the first CS presentation. **c** Location of cannula tips in the ACC. **d** Freezing to cues and 20 s pre CS presentation 24 h after direct fear conditioning and infusion of saline (*n* = 5) or muscimol (*n* = 7) indicate no differences in direct fear retention

Inactivating the vHPC prior to direct fear conditioning did not influence freezing during the direct fear conditioning session. Despite a significant fear conditioning cue × infusion group (muscimol or saline) interaction (*F*(2,16) = 3.89, *P* = .042), the overall between subjects effect of drug did not quite reach significance (*F*(1,8) = 3.99, *P* = .08) (Fig S5a). An independent samples *t* test comparing freezing during the 20 s prior to the first cue of long-term memory tests on day 3 revealed that rats that received muscimol infusions into the ventral hippocampus prior to fear conditioning froze significantly less to the context just before cue presentation 24 h after cued fear conditioning than rats infused with saline into the same region (*t*(4.45) = 3.26, *P* = .026). The same rats also froze significantly less to the cues presented during long-term memory (*t*(8) = 2.43, *P* = .043) although this could be explained by the reduced contextual freezing (Fig. 9b).

Inactivating the ACC prior to direct fear conditioning did not influence later expressions of freezing to the cue (*t*(10) = .09, *P* = .93) or the context (*t*(10) = .26, *P* = .8) (Fig. 9d). There was neither a cue by infusion group interaction (*F*(2,20) = .077, *P* = .93) nor a between subjects effect of infusion group on freezing during direct fear conditioning (*F*(1,10) = 1.143, *P* = .31) (Fig S5b).

## Discussion

Using fear conditioning by proxy as a means to measure social fear learning, we found that the dominance relationship between the demonstrator and observer is essential in predicting the success of social fear transmission. Although this form of learning overlaps with traditional Pavlovian fear conditioning, there are unique brain regions recruited that may support the use of this paradigm in detecting behavioral changes in learning ability associated with social cues not relevant in direct fear conditioning.

### Intra-cage play behavior as an indicator of dominance status correlates with socially learned fear responses

Consistent with Kavaliers et al.’s (2005) research on the social transmission of fear of biting flies, we also found that subordinate animals displayed an increased fear response after a social learning session with a dominant demonstrator. Here, S1 FCbP rats froze significantly more after fear conditioning by proxy with either the D rat or S2 rat than S1 rats that were not fear conditioned but S2 rats only showed socially acquired freezing after fear conditioning by proxy with the D rat. The dominant rat did not freeze to the cues after fear conditioning by proxy with either subordinate.

Studies of dominance in laboratory rats often yield conflicting results as the method used to determine dominance, degree of captivity, and even the very definition of dominance varies greatly between laboratories. Less naturalistic settings and/or more domesticated animals can lead to less pronounced aggressive behaviors (Adams 1980; Boice 1973; Calhoun 1963; Lore and Flannelly 1981; Robitaille and Bovet 1976), making observable asymmetries in such aggression difficult to detect. For the purpose of the social analysis performed in this set of experiments, dominance was defined here as an asymmetry in social behavior between familiar rats (Adams and Boice 1989; Bernstein 1981) that functions to prioritize access to resources when they are limited. It is important to point out that even in more aggressive strains of laboratory rats, true agonistic behavior constitutes a relatively rare event with even very long sampling intervals of behavior indicating that less than 0.5 % of the time in the colony is spent engaged in any overt fighting behavior (Adams and Boice 1989; Blanchard and Blanchard 1990, 1988). The majority of these offensive and defensive social behaviors are a form of “ritualized” aggression (Lorenz 1966) that function to maintain the social hierarchy (Scott 1966). To assess the effect of dominance in the typically docile strain of Sprague–Dawley rats, we used a definition of social dominance posed by Pellis and colleagues (Pellis et al. 1993) describing asymmetries in the reciprocal social behaviors (e.g., play fighting) within a cage of three young adult laboratory bred rats.

In order to validate asymmetrical social behavior as a measure of functional dominance, a subset of animals underwent both social behavior tests as well as competition for sweetened milk. It was found that the rat designated as the dominant rat through social observations also spent significantly more time drinking sweetened milk than the two subordinate rats.

The social information from dominance tests allowed us to determine which behaviors were essential for predicting later fear behavior and which were redundant in their contribution to social learning. We found that social contact occurring during the cues of fear conditioning by proxy remained the strongest predictor of LTM freezing in line with our earlier studies in males (Bruchey et al. 2010). Overall nape contacts initiated by the FCbP rat toward the FC rat and high evasion of FC rat in response to nape contacts, both perhaps indicators of dominance status with subordinate rats counter attacking in response to nape contacts less than dominant rats, uniquely contributed a moderate, but significant amount to freezing. These results suggest that the magnitude of the behaviors relevant to the maintenance of a social hierarchy is important for interpreting cues essential for vicarious fear learning. Future studies will investigate whether these same social behaviors are indicative of social fear transmission in female rats, where the role of dominance in the colony as well as behaviors that manifest in response to threat can differ from males (Adams and Boice 1983; Ziporyn and McClintock 1991) and may require colony manipulations that include maternal activity (Adams and Boice 1983). Work on female mice does indicate a similar role of submissive behaviors in prolonged social learning of food preference (Clipperton et al. 2008).

### Negative-affect ultrasonic vocalizations correlate with social fear transmission

Indicators of reciprocal relationships between an FC-FCbP pair of rats such as high percentage of the FCbP rat initiating nape contact with the FC rat and low levels of counters against nape contacts initiated by FC rat seem to be related to duration of 22 kHz calls during the fear conditioning by proxy session. These behaviors are complementary and suggest that the FC rat is more likely to emit alarm calls for a rat that it has a previously established a reciprocal affiliative social relationship with (increased attempts to initiate play with the target of the playful contact less likely to display a facing attack in response). It may not always be advantageous to alert a neighbor to danger. Although these calls are inaudible to many predators (e.g., humans, birds) (Schwartzkopff 1955), a number of additional predators of rats can hear vocalizations in the ultrasonic range (e.g., cats, dogs) (Sales and Pye 1974) and such emission could alert them to the location of its prey (Litvin et al. 2007). These data suggest that 22 kHz calls may represent an underlying motivation to socially transfer threat information to a conspecific (Blanchard et al. 1991; Brudzynski and Chiu 1995), similar to alarm calls studied in vervet monkeys (Seyfarth et al. 1980) and meerkats (Clutton-Brock et al. 1999), but only when the social relationship is characterized by high play initiation and low levels of facing attack as a defensive response. Together these results, support ideas proposed by others that social factors in some mammals can determine who is responsible for alerting others in the colony to potential dangers (e.g., dominance in rats (Blanchard et al. 1991), social complexity in marmots (Blumstein and Armitage 1997), or sentinel assignment in meerkats (Clutton-Brock et al. 1999).

The number of positive-affect 50 kHz calls emitted during the social learning session was negatively correlated with freezing displayed during tests for fear retention the following day. The social nature of these higher frequency 50 kHz calls (Knutson et al. 2002) suggests that they are produced in situations very similar to the fear conditioning by proxy paradigm (e.g., proximity to a conspecific) but likely do not code for information relevant to the impending threat. Interestingly, our data here indicate that 50 kHz vocalizations may impede the social transfer of associative fear information between rats, possibly due to their conflicting emotional valence (anticipation of reward) with the fear behavior displayed by the FC rat. However, because both the FC and FCbP rats were recorded from the same chamber on day 2 and FC, FCbP, and No FC rats all emitted 50 kHz frequency vocalizations on day 3, no conclusions can be made about which rat was vocalizing on day 2.

### Elevated serum corticosterone levels in avoidant subordinates and rats with direct fear experience

Subordination in rats that do not readily accept their subordinate roles (e.g., S2 rats) may result in animals that fail to properly regulate the stress response in novel situations (Chapman et al. 1969; Ely and Henry 1978) consequently negatively impacting the overall health of the animal. Although basal levels of hormones were not measured here, the heightened levels of corticosterone in No FC S2 rats are in line with this concept of subordination stress in avoidant rats. Stress can influence the formation of a dominance hierarchy, mostly by affecting the social behaviors of subordinate animals (Cordero and Sandi 2007; Timmer et al. 2011; Timmer and Sandi 2010). While the studies performed here were conducted in established cages, it is possible that dominant and subordinate animals react differently to the stress of the fear conditioning by proxy paradigm thereby influencing the fear response displayed the following day. Additionally, exogenous corticosterone has been found to facilitate social learning of food preferences (Choleris et al. 2013), further supporting a role for corticosterone in social fear learning.

It is important to note that vicarious fear learning in the fear conditioning by proxy paradigm did not result in significantly higher circulating corticosterone levels compared to rats that were only exposed to the CS. When rodents observe conspecifics in distress, their own physiological stress response is typically activated, which is suspected to play a role in aversive learning through observation (Kavaliers et al. 2003). Our results suggest that in the fear conditioning by proxy paradigm employed here, exposure to a freezing conspecific is not inherently more stressful to the animal than hearing a novel tone.

Although significant, the relationship between corticosterone and freezing was not strong suggesting that the stress response observed here is only partially concordant with the behavioral response measured here (e.g., freezing). As a whole, all of the rats in this experiment had very high levels of corticosterone after exposure to the cues during LTM (48 h after direct fear conditioning or 24 h after social fear conditioning), even rats that were not previously fear conditioned (No FC rats). These levels are consistent with those seen after exposure to acute stress or novelty (Marin et al. 2007), which seems to be induced by this paradigm, making interpretation of perhaps ceiling levels of hormone difficult.

### Neural mechanisms of fear conditioning by proxy

The neural processes underlying fear conditioning by proxy were evaluated in parallel with those involved in direct fear conditioning using c-Fos immunohistochemistry and temporary regional inactivation with muscimol. Quantification of c-Fos protein was used a surrogate marker of neuronal activity (Dragunow and Faull 1989; Hoffman et al. 1993) to provide some initial insight into the possible regions activated by social fear learning in the fear conditioning by proxy paradigm (experiment 2). Regions of importance identified through the c-Fos experiment were then targeted with muscimol to determine their sufficiency for the relevant behaviors of interest.

Intracranial infusions of muscimol or saline 20 min prior to fear conditioning (experiment 4) or fear conditioning by proxy (experiment 3) indicated that the anterior cingulate cortex, but not the ventral hippocampus, is necessary for the social transmission of fear in this paradigm. Conversely, and consistent with other studies (Bannerman et al. 2003; Biedenkapp and Rudy 2009; Maren 1999; Maren and Holt 2004; Richmond et al. 1999), inactivation of the ventral hippocampus prior to direct fear conditioning resulted in freezing deficits when tested later.

Inactivating the anterior cingulate cortex with muscimol prior to direct fear conditioning did not influence later expressions of freezing. Despite the ACC’s contributing role in direct emotional learning, it is not a required part of the fear conditioning pathway but is necessary for socially learned fears.

The importance of the ACC in maintaining and evaluating attentional resources, especially in emotionally conflicting situations, such as the presence of a threat, is evidenced in rodents by the region’s role in attentionally demanding tasks (Bussey et al. 1997; Muir et al. 1996), including visual observational fear conditioning (Jeon et al. 2010). Human research parallels these findings, with the ACC implicated in guiding response selection during conflict (Botvinick et al. 2004; Pardo et al. 1990; Posner and Petersen 1989). Together these studies indicate that the mechanisms involved in social fear learning in rodents may overlap across species, including rats, mice, and even humans. In the fear conditioning by proxy paradigm presented here, evaluating the salience of cues found during environmental exploration of a potentially threatening situation (FC rat behaving fearfully) may be mediated by the ACC as a means of monitoring their surroundings and assessing threat (Fiddick 2011).

One interpretation is that the fear conditioning by proxy paradigm is very similar to Pavlovian fear conditioning with the exception that the vicarious nature requires the rat to evaluate the many available sensory and social cues to a greater degree than direct CS-US pairing would. With direct fear conditioning, previous presentations of the CS are followed by an aversive event and learning depends on reflexive responding, a form of learning that is dependent on the amygdala and ventral hippocampus. In vicarious conditioning, no reflex has occurred, therefore when a relatively novel event occurs (placement into a chamber and presentation of a noise), the animal must first appraise the situation, in order to respond.

Assuming that the ACC is necessary for attentional selection (Muir et al. 1996; Bussey et al. 1997), inactivating this region causes previously relevant social cues (e.g., D rat behaving fearfully) to lose saliency because the animal is no longer selectively attending to them. We suggest, in line with human and rodent research, that the anterior cingulate cortex is essential for this form of threat appraisal and consequently learning through vicarious experience.

## General discussion and conclusions

In rats, most early studies of social learning surrounded the social transmission of food preference. These experiments typically take one of two forms depending on when the stimulus (e.g., food) is present. In the first form, rats observe a conspecific consuming food of a specific flavor and are tested for their preference for that flavor. In the second form, rats interact with a conspecific after consumption of a specific flavor and are then tested for their flavor preference. Considerable research has shown that, in both forms, rats will show a preference (at least initially) to ingest a food type that another rat has already ingested (Galef and Kennett 1987; Galef et al. 1984; Galef and Wigmore 1983; Posadas-Andrews and Roper 1983; Richard et al. 1987; Strupp and Levitsky 1984; Valsecchi and Galef 1989).

In addition to an “observer” subject visually witnessing a “demonstrator” animal perform a task, these paradigms of the social transmission of food preference also allow the animal to incorporate auditory, olfactory, and in some cases gustatory and tactile information. In addition to foraging for food, rodents must be on the constant look out for predators, providing researchers with another biologically salient means to study social learning. However, to date, most studies of vicarious fear learning do not allow subjects to have physical contact during the social fear learning session.

In the experiments presented here, we explore the neural and hormonal mechanisms that underlie a novel modified demonstrator–observer paradigm that allows for free interaction between subjects and emphasize the importance of social dominance in the social transmission of fear. Importantly, we show that differences in the social behaviors of subordinate animals must be considered when considering the relationship between groups of rats on social fear transmission.

The behaviors of the subordinate rats may represent different coping strategies to deal with subordination (Schenkel 1967; Von Holst et al. 1983), with S1 actively coping by establishing a tolerable relationship with the dominant and S2 taking a more passive approach of avoidance. In manipulations of the dominance hierarchy with triads of rats with the same social designations used here, Pellis and colleagues found that removal of the dominant rat from the cage usually resulted in the S2 rat becoming dominant (Pellis et al. 1993). In experiment 1, we show that S1 rats learn equally from either the D rat or the S2 rat, but the S2 rat only learns from the D rat. Together, these results suggest that vicarious fear learning only occurs when higher “ranked” rats in the dominance hierarchy display cues relevant to their direct fear conditioning experience. However, when tested for dominance through competition for limited access to sweetened milk, there was no discernible rank among the two subordinates. Given that only one rat could drink from the container at a time, it is possible that drinking rats in subordinate rats may diverge if the dominant rat were removed or if animals were allowed longer access. Future research will further expand on how social subordinates prioritize access to limited resources. Within the framework of behavioral responses to threat, dominant rats may transiently behave more submissively and subordinate rats more dominantly (Blanchard and Blanchard 1990), which may influence the transmission and expression of fear behavior in individual rats.

Our results suggest that fear conditioning by proxy draws on some of the same processes as fear conditioning through direct experience (e.g., LA involvement). However, learning through vicarious experience may require a more elaborate process of threat evaluation and interpretation of how to respond to a novel stimulus, a process that engages and is dependent upon the anterior cingulate cortex.

Determining the social roles of rats, and how their acceptance, or the stability, of said roles influences learning strategies and stress responding, may help us develop more translationally relevant models of behavior. The research presented here demonstrates that the intricacies of play fighting among related male rats are crucial to the social transmission of fear and underscores the importance of integrating observations of social relationships in the interpretation of behavioral data acquired from social species, including the commonly used laboratory rat.

## Electronic supplementary material

Below is the link to the electronic supplementary material.

Supplementary material 1 (DOCX 7158 kb)
