# Supplementary material for: Dominance status predicts social fear transmission in laboratory rats
Source: Anim Cogn. 2016 Jul 13;19(6):1051–69. doi: 10.1007/s10071-016-1013-2 (PMC5054054; doi:10.1007/s10071-016-1013-2)
Supplement: Supplementary file 1 — Supplementary material 1 (DOCX 7158 kb) [file 10071_2016_1013_MOESM1_ESM.docx]

**Dominance Status Predicts Social Fear Transmission in Laboratory Rats**

Carolyn E. Jones and Marie-H. Monfils

**Supplementary Methods** p2-p6

**Supplementary Figures and Tables** p7-p14

**Contact Information:**

Carolyn E. Jones, PhD

[carolyn.eagan@gmail.com](mailto:carolyn.jones@utexas.edu)

The University of Texas at Austin

Department of Psychology

108 E. Dean Keeton Stop A8000

Austin, TX 78712-1043

**Supplementary Methods**

**Social Determination of Dominance - Social Interactions**

Not all cages had a clear asymmetry in play behavior, defined by one rat receiving a disproportionate share of nape contacts within a cage, at the time of dominance tests, with only approximately 64% of cages tested displaying such an asymmetry between the three rats. Approximately 20% of the cages tested initiated nape contacts equally to all cage-mates and therefore displayed no asymmetry in nape contact (each member of the triad received approximately 33% of the nape contacts). Of these cages, all rats tended to display equal rates of counterattacks and complete rotations to supine in response to nape contacts. A small portion of the cages tested (<10%) had 2 rats that were equally contacted and both of these rats counter attacked both each other and the subordinate at high rates (**Fig. S1)**. The third rat was clearly subordinate, initiating the majority of nape contacts and responding with rotation at very high rates. These cages were considered to have 2 dominants and 1 subordinate (**Fig. S1)**. An equally small portion of cages tested (<10%) had a clear dominant rat that contacted the 2 subordinate rats equally with both subordinates behaving similarly in response to contact (**Fig. S1)**.

**Competitive Dominance**

Cages were first run through play behavior tests and a D, S1, S2 hierarchy status was assigned within each cage using the nape contact/defensive response method described previously. One week later competitive dominance was determined by measuring drinking out of a conical ceramic food dish (bottom diameter=5cm; top diameter=3.75cm) filled with 57mLs of sweetened condensed milk (Eagle™) diluted with water (2 parts water to 1 part sweetened condensed milk). This food dish was designed so that only one rat could drink out of it at a time.

*Habituation:* Prior to competition, rats were habituated over four days to the food dish and the sweetened milk. On the first two days of habituation, all rats were single housed and the ceramic dish filled with sweetened milk was placed in their home cage for a period of 6 hours. This single habituation was performed to ensure that all rats overcame their neophobia of the novel dish and learned that the dish contained a desired treat. All rats eventually drank the sweetened milk from the ceramic dish on both days of single housed habituation. On days 3 and 4 of habituation, rats remained housed in triads, and the dish with sweetened milk was placed in the home cage for 3 hours each day. At the end of this 3 hour window, the dish was removed from the home cage. All of the milk was consumed during this habituation period.

*Competition:* Two sessions of sweetened milk competition were recorded. The ceramic dish, filled with sweetened milk, was placed in the far end of the same recording bins used for play behavior observations. All three rats from a triad were placed in the bin at the end opposite the food dish and recorded for 7 minutes. The amount of time that each rat spent drinking was measured in 60-second bins. For each 60 second bin, the total time all the rats spent drinking was calculated and each individual rat’s time drinking was divided by the time spent drinking by all rats. This created a normalized percent drinking time for each rat indicative of monopolization of the resource. The percent drinking time for each rat was averaged across the two competition sessions and analyzed as a function of play behavior dominance assignment.

**Ultrasonic Vocalization Analysis (Experiment 1)**

In accordance with previous research in our lab (Shumake et al. 2014) and others (Burgdorf et al. 2009; Burgdorf et al. 2005; Schwarting et al. 2007), there was a lot of inter-rat variation in vocalization, and the majority of rats did not emit any vocalizations. Both negative-affect (22kHz frequency range) and positive-affect (50 kHz frequency range) vocalizations were measured in the time points immediately surrounding cue presentation (20 seconds prior to CS onset, during the 20 seconds that the CS played, and 20 seconds post CS) as well as throughout the entire duration of the session. Only two rats vocalized in any of the time points surrounding presentation of the cue and they were consequently vocalizing for the majority of the session (data not shown). As a result, and because all rats were in the chambers for equal amounts of time, vocalizations were analyzed over the entire behavioral session without regard for CS presentation.

**Histology (Experiment 2)**

Free-floating sections from an entire series were rinsed in 0.1M phosphate buffered saline (PBS) to remove cryoprotectant. Sections were incubated in 0.03% H_2_O_2_ in PBS for 15 minutes to quench endogenous peroxidase, washed three times (10 minutes each wash) in PBS, and then blocked in 6% normal goat serum in PBS combined with 0.3% triton-x (PBST) for 1 hour at room temperature. They were immediately transferred from the blocking solution to an incubation in primary antibody to c-Fos raised in rabbit (sc-52, Santa Cruz Biotechnologies, Inc., Santa Cruz, CA, USA) at 1:10000 in PBST with 3% normal goat serum for 72 hours at 4° C. After incubation in the primary antibody, sections were washed and incubated in biotinylated goat-anti-rabbit secondary antibody (Vector Laboratories, USA) at a concentration of 1:250 in PBST with 3% normal goat serum for 1 hour. Sections were again washed three times for 10 minutes in PBS prior to incubation in avidin-biotin complex (ABC Elite kit; Vector Laboratories, USA). ABC mix was prepared according to kit instructions and allowed to mix for 30 minutes prior to the 60-minute incubation at room temperature. After three rinses, sections were developed in chromogen solution containing 0.02% 3,3’ diaminobenzidine tetrahydrochloride (DAB; Sigma-Aldrich, St. Louis, MO, USA) in phosphate buffer with .01% hydrogen peroxide for 3-5 minutes. Nickel sulfate (.08%) was added to the DAB solution to result in a blue-black staining. Sections were mounted onto slides and left to dry overnight. Once dry, slides were dehydrated in an ethanol gradient, cleared in xylene, and cover slipped with Permount.

**Surgical Procedure (Experiments 3 and 4)**

Rats were anesthetized with isoflurane and mounted in a stereotaxic frame with lambda and Bregma in the same horizontal plane. The scalp was incised and retracted. Small burr holes were drilled for placement of 4 stainless steel jewelers screws and bilateral guide cannula targeting the determined brain region based on coordinates from the brain atlas of Paxinos and Watson (2009).

*Ventral hippocampus*: The coordinates for the ventral hippocampus were: 5.1 mm posterior from Bregma, and 4.6 mm lateral to midline and bilateral guide cannulae (26 gauge, Plastics One) were lowered 6.7 mm ventral from dura. Dental acrylic (Dentsply, USA) was applied to the skull and dummy cannulas cut flush with the guide cannulae (33 gauge, Plastics One) were inserted into the guide cannulas. All rats were injected with .03mg/kg of Buprenorphine (IP) at the completion of the surgery to reduce any post-operative pain. Rats were checked twice daily during recovery and dummy cannulas were replaced as necessary. In experiment 3, After 5 days, rats were rehoused into triads and day one of the fear conditioning by proxy paradigm began 24 hours later. In experiment 4, rats remained single housed.

*Anterior cingulate:* A 5-mm stainless steel guide cannula (26 gauge, Plastics One) was implanted bilaterally at a 16° angle into holes drilled at 1.8mm anterior from Bregma and 1.6mm lateral to the midline and lowered 1.6 mm ventral from dura.

**Muscimol Infusions (Experiments 3 and 4)**

In experiment 3, rats were housed in triads but only the FCbP rat in each cage received an infusion of either saline or muscimol. In experiment 4, rats were single housed and no social learning tests were performed. In experiment 4, all rats were infused with either muscimol or saline prior to direct fear conditioning.

*Ventral hippocampus*: Twenty minutes prior to either direct fear conditioning (experiment 4) or fear conditioning by proxy (experiment 3) rats received bilateral infusion into the ventral hippocampus through an injection cannula (33 gauge, Plastics One) that extended 1 mm below the guide cannula. Polyethylene tubing connected the injection cannula to a 10 μL Hamilton syringe mounted in a microinfusion pump that delivered either muscimol (1μg/μL dissolved in saline) or vehicle (0.9% saline) for one minute at a rate of 0.5 μL/min (0.5 μg/0.5μL/side) in order to limit drug diffusion within 1mm beyond injection site (Zhang et al. 2014). Injectors were left in place for an additional minute to allow complete diffusion of the drug into the ventral hippocampus. This dose of muscimol (0.5μg in 0.5μL saline) is consistent with other research on both cued and contextual fear conditioning targeting the ventral hippocampus and has been found to result in significant behavioral impairments in spatial tasks as well as contextual fear conditioning paradigms that require the hippocampus without overall locomotion decreases seen at higher doses (e.g. 1.0μg/0.5μL side) (Hobin et al. 2006; Zhang et al. 2014).

*Anterior cingulate:* Twenty minutes prior to either direct fear conditioning (experiment 4) or fear conditioning by proxy (experiment 3) rats received bilateral infusion into the anterior cingulate through an injection cannula (33 gauge, Plastics One) that extended 0.5 mm below the guide cannula. The injection cannula was attached by a polyethylene tube (PE 20, Plastics One) to a 10μL Hamilton syringe. A microinfusion pump (PhD 2000; Harvard Apparatus, USA) infused a volume of .2μL per side of either muscimol (1 μg/μL dissolved in saline) or vehicle (0.9% sterile saline) over 2 minutes and the cannulas were left in place for another 2 minutes after completion. In the anterior cingulate cortex, this was equal to a total muscimol infusion of 0.2μg/0.2μL/side determined from previous research on the affects of muscimol infusion into the anterior cingulate and other prefrontal regions on fear learning (Bissière et al. 2008; Liu et al. 2009; Sierra-Mercado et al. 2011).

**Histology for cannula verification**

At the conclusion of the behavioral tests rats were perfused and brains stored in 4% paraformaldehyde (PFA) for 24-48 hours before transfer to sucrose for cyroprotection. Brains were sectioned on a freezing microtome at 40um and sections relevant to the cannula location were stained with cresyl violet to enhance visibility of neural landmarks. Histological verification of cannula placement was performed by a trained observer, blind to experimental condition, based on the atlas of Paxinos and Watson (2009).

**Corticosterone Analysis**

The corticosterone EIAs had an assay sensitivity of 150pg/mL. The mean intra-assay variability for all samples on the plate was less than 5%. The inter-assay variability was 10.2% and groups were distributed across plates. Sample values were not included in analysis if the %CV associated with that sample value was greater than 10%. Three values were discarded for this reason.

Values for the two dilutions for the control sample were plotted for each assay to determine both intra- and inter-assay variability as a measure of quality control. Although overall both inter- and intra- assay variability was low (%CV<10%), intra-assay variance was significantly lower for all control samples and dilutions than inter-assay variance (all ps<.05). In order to compare values across different plates, a grand mean calculated for control samples read from the dilution that best matched the linear range of the standard curve (a logarithmic curve of percent bound vs. corticosterone concentration of known concentrations provided with each kit) from each plate was used to create a scaling factor for each plate based on that plate’s sample control reading. This scaling factor was then applied to each sample’s corticosterone reading to create a normalized value that was used for analysis.

**Cell Quantification and Analysis**

C-Fos positive cells were counted from 6 brain sections for each rat. If six viable sections were not available, subject data was not included if less than 5 sections were viable for imaging in a given region. Sections were chosen according to the brain atlas of Paxinos and Watson (Paxinos and Watson 2009) and counting frames were aligned as detailed in Fig 5a. Images were taken using a Zeiss AxioCam MRm digital camera at 10x magnification. Cells were counted using ImageJ software (NIH) for Mac. Care was taken to match sections for each brain and regions were sampled from both the left and right hemispheres. Total cell counts/.1mm^2^ were calculated for each animal and means were statistically analyzed using ANOVA with significant main effects followed up by post-hoc Tukey mean comparisons.

Immunopositive nuclei were counted by an observer blind to experimental group in the CG1 region of the anterior cingulate cortex, the lateral nucleus of the amygdala (LA), and the CA1 region of the dorsal hippocampus using a fixed counting frame in a given structure as described below.

Anterior Cingulate Cortex: Cells in the CG1 region of the anterior cingulate cortex were sampled from sections corresponding to Bregma +3.00, +2.28, and +1.56. Immunopositive cells were counted from 3 alternating circular frames aligned along the diagonal of the image starting at the genu of the corpus callosum (Fig 1b).

Lateral Amygdala: Cells in the lateral nucleus of the amygdala were sampled from sections corresponding to Bregma -2.76, -3.00, and -3.24 (Paxinos and Watson 2009). Immunopositive cells were counted from three identical cicular counting frames (total area = .072 mm^2^) fitted within the boundaries of the lateral amygdala (Fig 1b).

Dorsal Hippocampus: Cells in the dorsal hippocampus were sampled in in the CA1 and dentate gyrus (DG) subregions from sections corresponding to Bregma -3.24, -3.48, and -3.72. An image was taken from each section by aligning the top left corner of the frame with the apex of the pyramidal layer of CA1. The imaging frame for the DG was taken in a similar manner by aligning the edge of the image with the apex of the granular layer of the DG. Counting frames consisted of identical circles placed within the visible region of interest and cells were counted from alternating circles (CA1: Fig 1b). The total area sampled for the CA1 was .019mm^2^ and the total area sampled for DG was .039mm^2^.

Ventral Hippocampus: Cells in the ventral hippocampus were sampled in the CA1, CA3, and CA2 subregions from sections corresponding to Bregma -4.7, -4.9, -5.2. Counting frames consisted of identical circles placed within the visible region of interest and cells were counted from alternating circles (Fig 1b).

**Fig. S1 Frequency of social hierarchy subtypes in triads of males**

The majority of rats had a clear dominance hierarchy with D, S1, and S2 behavioral phenotypes. A subset of rats all contacted each other at equal frequencies and displayed similar defense responses to contacts and a smaller percentage had either one dominant rat with equal subordinates or two seemingly dominant rats with only one subordinate. Only rats with a clear dominance hierarchy were used in the present experiments.

**Fig S2. Monopolization of sweetened milk by social behavior dominance groupings**

Rats that received a disproportionate share of nape contacts within a cage of triads (D rats) also spent the most amount of time drinking from a container filled with sweetened milk compared to the subordinate rats. Error bars +/- SEM.


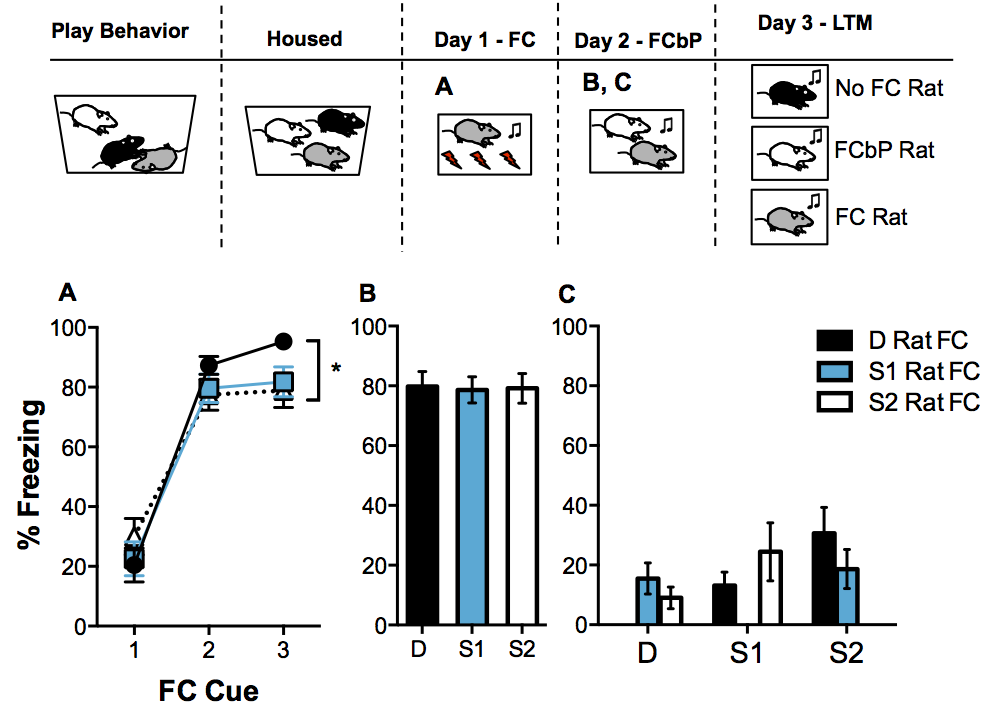


**Fig S3**. **Freezing to cues on day 1 and day 2 in FC rats and FCbP rats**

A) There were marginal, but significant differences in freezing during the final CS+US pairing of the direct fear conditioning session on day 1, (one-way ANOVA, *F*(2,57)=3.55, *P*=.035) with D rats freezing more than S2 rats during the third CS presentation of fear conditioning (post-hoc Tukey, *P*=.038) (fig. S3a). The difference in freezing between dominant and subordinate 2 rats during the last cue of fear conditioning, although significant, was minor given that all rats froze at very high levels at the end of fear conditioning (dominant rats mean=.95, SD=.07; subordinate 2 rats mean=.79, SD=.26) and B) there were no differences in direct fear retention when tested the following day. C) Freezing in FCbP rat on day 2 revealed no significant effect of either dominance of either the observer or demonstrator (all ps>.05). Error bars +/- SEM.

**Fig S4.** **50kHz calls during fear conditioning by proxy paradigm**

A) Sample spectrograph of 50kHz vocalizations. Frequency histograms of the number of subjects that emitted positive-affect vocalizations in the 50+kHz range during B) fear conditioning on day 1, C) fear conditioning by proxy on day 2, and D) long term memory tests on day 3 indicate that more rats vocalize in the 50kHz range than the 22kHz range but a large number of rats still do not vocalize at all. E) of the rats that do vocalize (more than 1 vocalization on day 2; n=27), the total number of 50kHz calls during the FCbP session on day 2 was negatively correlated with freezing displayed by the FCbP rat on day 3 (*P*<.05).

**Fig S5. Muscimol on freezing during FC in experiment 4**

Freezing during direct fear conditioning after infusions of either saline (open circles) or muscimol (blue diamonds). There were no significant differences in freezing during direct fear acquisition after infusion into either the ventral hippocampus (A) or the anterior cingulate cortex (B). Error bars +/- SEM.

|  | FCbP Rat | FC Rat | No FC Rat |
| --- | --- | --- | --- |
| Cage Combo 1  (n=12) | D | S1 | S2 |
| Cage Combo 2  (n=11) | D | S2 | S1 |
| Cage Combo 3  (n=10) | S1 | D | S2 |
| Cage Combo 4  (n=10) | S1 | S2 | D |
| Cage Combo 5  (n=9) | S2 | D | S1 |
| Cage Combo 6  (n=9) | S2 | S1 | D |

**Table S1.** **Possible Cages for Each Dominance Combination in Experiment 1**

Rats from each cage combination are identified by indicating dominance status of the rat in question immediately followed by the rats fear conditioning assignment, the dominance of the FC rat in the cage comes after a dash (e.g. “D FCbP-S1 FC” refers to the FCbP rat in cage combo 1 in the first cell).

**Table S2. Social Behaviors Scored During Dominance Tests**

Descriptions of offensive and defensive behaviors tallied across three social interaction sessions.


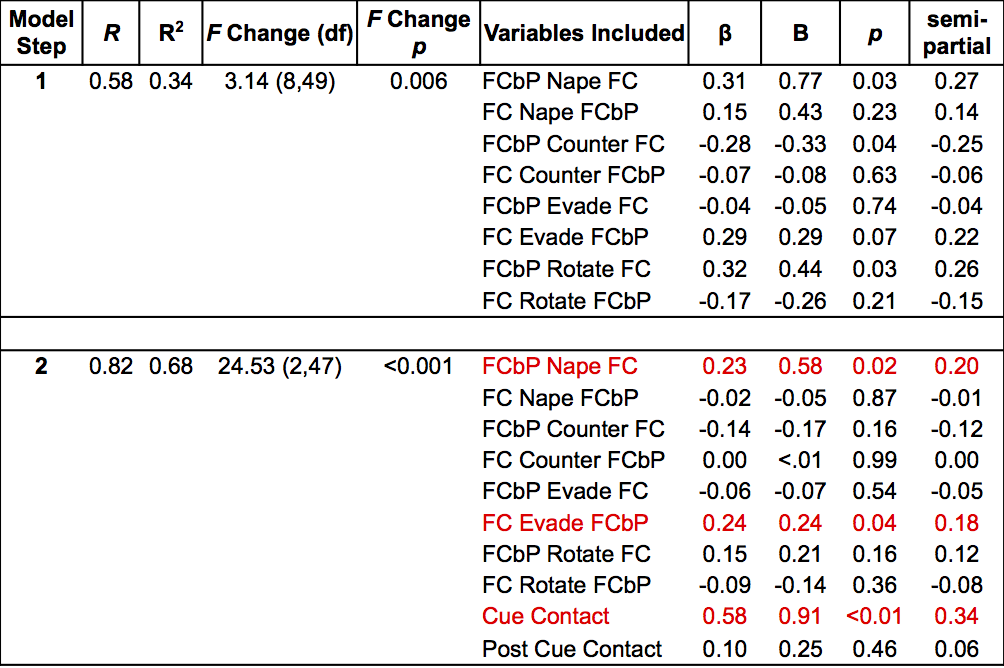


**Table S3**. **Hierarchical Regression Model of FCbP rat Freezing on Day 3**

Each step of the model resulted in a significant R^2^ change and the final model included: % of total nape contacts within a cage that FCbP rat nape contact FC rat (p=.02), likelihood of FC rat to respond to nape contacts with evasion (p=.04), and % duration of cue that was spent engaged in social contact on day 2 (p<.01) each significantly contributed to variance in freezing on day 3 displayed by the FCbP rat. Predictor variables in red text indicate significant (p<.05) contribution to the model change.


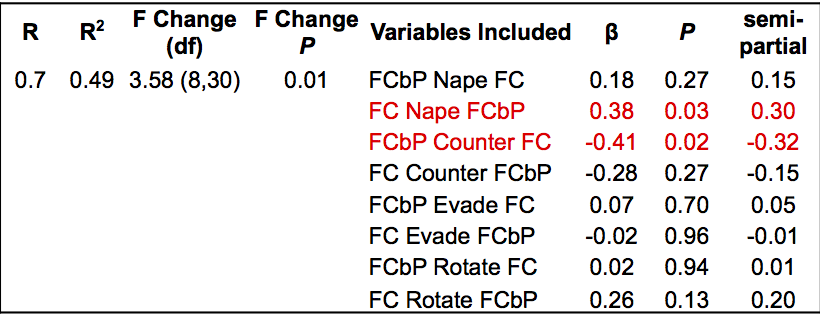


**Table S4**. **Regression model of play behavior predictors of 22kHZ USVs**

Predictor variables in red text indicate significant (p<.05) contribution to the model change. Increased percentage of friendly nape contacts within a cage that were initiated by the FC rat towards the FCbP rat and decreased likelihood that the FCbP rat responds by counterattacking the FC rat each significantly contributed to variance in duration of 22kHz USVs on day 2. However, a large amount of variance (approximately 50%) is still unaccounted for (R^2^=.49).

Bissière S, Plachta N, Hoyer D, McAllister KH, Olpe H-R, Grace AA, Cryan JF (2008) The rostral anterior cingulate cortex modulates the efficiency of amygdala-dependent fear learning. Biological psychiatry 63:821-831

Burgdorf J, Panksepp J, Brudzynski SM, Beinfeld MC, Cromwell HC, Kroes RA, Moskal JR (2009) The effects of selective breeding for differential rates of 50‐kHz ultrasonic vocalizations on emotional behavior in rats. Developmental psychobiology 51:34-46

Burgdorf J, Panksepp J, Brudzynski SM, Kroes R, Moskal JR (2005) Breeding for 50-kHz positive affective vocalization in rats. Behavior genetics 35:67-72

Hobin JA, Ji J, Maren S (2006) Ventral hippocampal muscimol disrupts context-specific fear memory retrieval after extinction in rats. Hippocampus 16:174-182. doi:10.1002/hipo.20144

Liu F, Zheng X-L, Li B-M (2009) The anterior cingulate cortex is involved in retrieval of long-term/long-lasting but not short-term memory for step-through inhibitory avoidance in rats. Neuroscience letters 460:175-179

Paxinos G, Watson C (2009) The Rat Brain in Stereotaxic Coordinates. 6th edn. Elsevier, London

Schwarting RK, Jegan N, Wöhr M (2007) Situational factors, conditions and individual variables which can determine ultrasonic vocalizations in male adult Wistar rats. Behavioural brain research 182:208-222

Shumake J, Furgeson-Moreira S, Monfils MH (2014) Predictability and heritability of individual differences in fear learning. Animal cognition 17:1207-1221

Sierra-Mercado D, Padilla-Coreano N, Quirk GJ (2011) Dissociable roles of prelimbic and infralimbic cortices, ventral hippocampus, and basolateral amygdala in the expression and extinction of conditioned fear. Neuropsychopharmacology : official publication of the American College of Neuropsychopharmacology 36:529-538

Zhang WN, Bast T, Xu Y, Feldon J (2014) Temporary inhibition of dorsal or ventral hippocampus by muscimol: distinct effects on measures of innate anxiety on the elevated plus maze, but similar disruption of contextual fear conditioning. Behavioural brain research 262:47-56. doi:10.1016/j.bbr.2013.10.044
